# Supplementary material for: Li7La3Zr2O12-co-LiNbO3 Surface Modification Improves the Interface Stability between Cathode and Sulfide Solid-State Electrolyte in All-Solid-State Batteries
Source: Membranes (Basel). 2023 Feb 9;13(2):216. doi: 10.3390/membranes13020216 (PMC9967944; doi:10.3390/membranes13020216)
Supplement: Supplementary file 1 [file membranes-13-00216-s001.zip › membranes-2102832-supplementary.pdf]

membranes

Article

$Li_7La_3Zr_2O_{12}$-co-$LiNbO_3$ Surface Modification Improves the Interface Stability between Cathode and Sulfide Solid-State Electrolyte in All-Solid-State Batteries

Shishuo Liang [1,2], Dong Yang [1], Jianhua Hu [1], Shusen Kang [2,*], Xue Zhang [3] and Yanchen Fan [4,5,6,*]

1 State Key Laboratory of Molecular Engineering of Polymer, Department of Macromolecular Science, Fudan University, Shanghai 200438, China
2 Sunwoda Electric Vehicle Battery Company, Shenzhen 518107, China
3 College of Resources and Environment, Jilin Agricultural University, Changchun 130118, China
4 Petro China Shenzhen Renewable Energy Research Institute Co., Ltd., Shenzhen 518000, China
5 CNPC Shenzhen New Energy Research Institute Co., Ltd., Shenzhen 518000, China
6 SUSTech Academy for Advanced Interdisciplinary Studies and Department of Materials Science & Engineering, Southern University of Science and Technology, Shenzhen 518055, China
* Correspondence: kshusen@163.com (S.K.); fanyc@sustech.edu.cn (Y.F.); Tel.: +15122485554 (Y.F.)

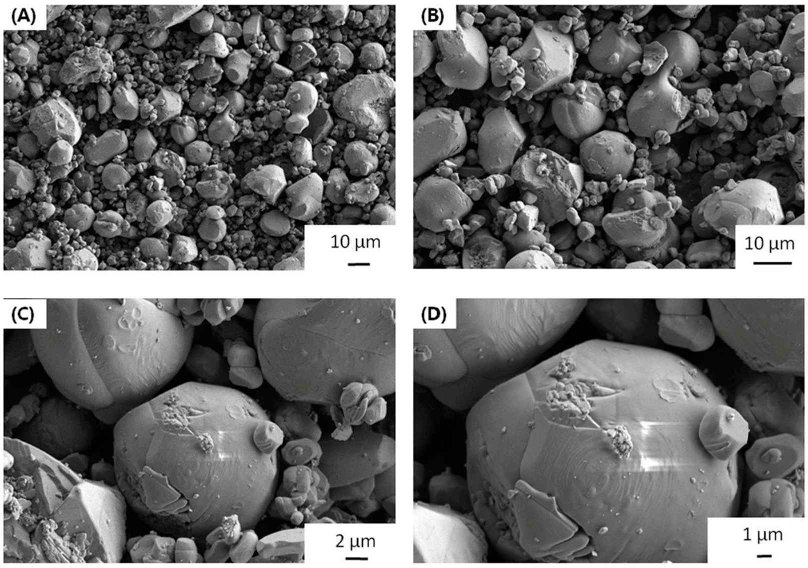

Figure S1. SEM image of LCO.

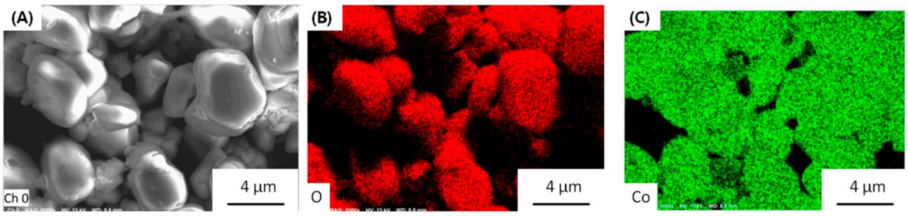

Figure S2. Elemental O, Co mapping of LCO.

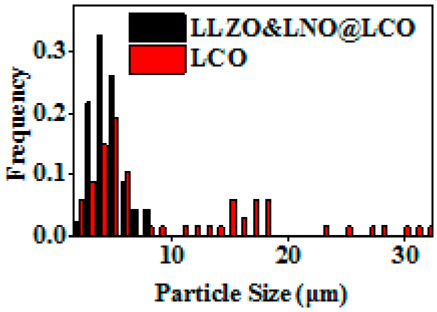

Figure S3. Particle size distribution of LLZO&LNO@LCO and LCO.
